# Supplementary material for: Intramuscular IL-10 Administration Enhances the Activity of Myogenic Precursor Cells and Improves Motor Function in ALS Mouse Model
Source: Cells. 2023 Mar 26;12(7):1016. doi: 10.3390/cells12071016 (PMC10093513; doi:10.3390/cells12071016)
Supplement: Supplementary file 1 [file cells-12-01016-s001.zip › cells-2251951-supplementary.pdf]

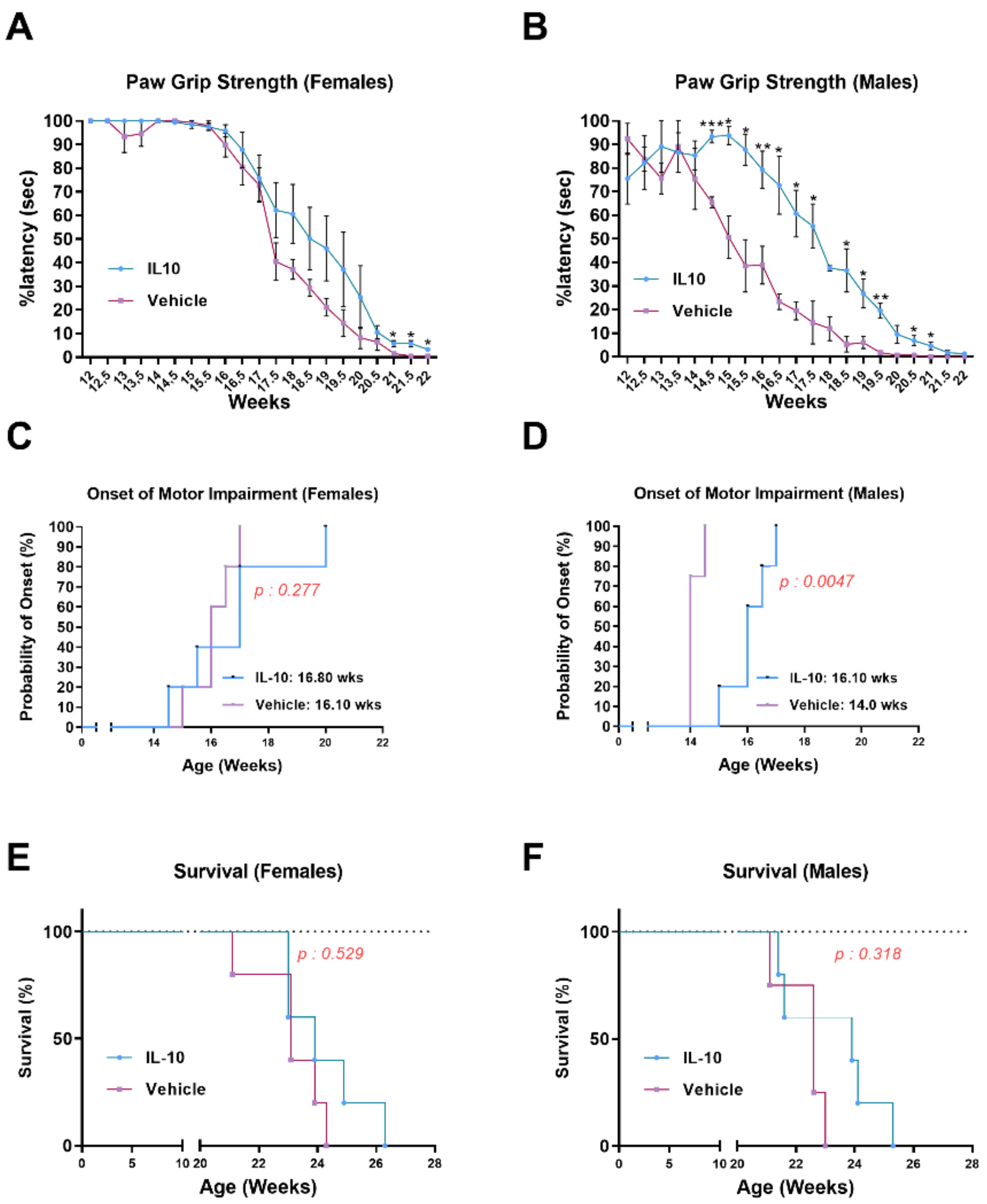

Figure S1. Disease progression and survival of C57-SOD1G93A female and male mice. (A, B) Paw Grip Endurance (PaGE) test for IL-10 (n = 5) and PBS (n = 4-5) treated C57-SOD1G93A females (A) and males (B) mice. The data are reported as mean ± SEM for each time point. $^{*}p < 0.05$, $^{**}p < 0.01$, $^{***}p < 0.001$ by repeated-measures ANOVA with Sidak's post-analysis. **(C, D)** IL-10-treated SOD1G93A male (C) but not female (D) mice had a delayed onset of motor impairment than PBS-treated mice. The *p* was calculated by the Mantel-Cox log-rank test. (**E, F**) IL-10-treated C57-SOD1G93A female (E) or male (F) mice did not show a significantly higher survival length than PBS-treated mice. The *p* was calculated by the Mantel-Cox log-rank test.

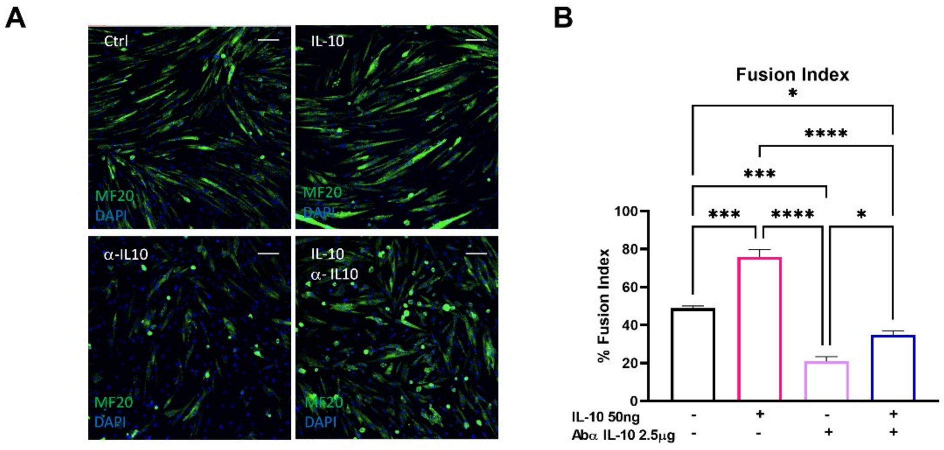

Figure S2. IL-10 improves the rate of muscle fibre differentiation of C57-SOD1G93A-derived SCs. (**A**) Representative confocal images showing the immunostaining for MF20-MyHC (green) and DAPI (blue) on primary C57-SOD1G93A SC cultures treated with 10ng/mL IL-10 and/or 2.5µg/mL AbαIL10 in DM for 48h. Scale bar =100µm. (**B**) The fusion index was calculated as (no. nuclei present in MyHC+ cells with two or more nuclei/no. myotubes). Data are reported by mean ± SEM from three independent experiments for each group. $^{***}p < 0.001$, $^{****}p < 0.0001$ by one-way ANOVA with Tukey's post-analysis.

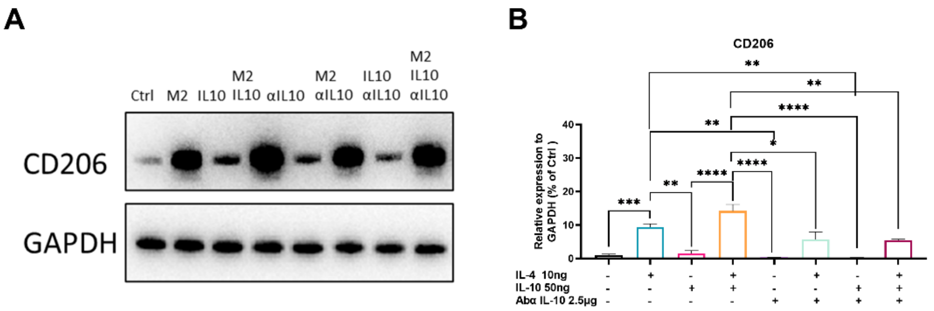

Figure S3. IL-10 enhanced MΦ polarisation to M2 anti-inflammatory fingerprint. (**A, B**) Representative immunoblot images (**A**) and densitometric analysis (**B**) of CD206 levels in C57-SOD1G93A-derived MΦ polarized in vivo for 24 hours to M2, with 10 ng/mL IL4 in the presence or absence of 50ng/mL IL-10 and/or 2.5µg/mL AbαIL10. The data are reported as the percentage of untreated cells (mean ± SEM) of six independent experiments for each group. $^{*}p < 0.05$, $^{**}p < 0.01$, $^{***}p < 0.001$, $^{****}p < 0.0001$ by one-way ANOVA with Tukey's post-analysis.
